# Connexin40 controls endothelial activation by dampening NFκB activation

**DOI:** 10.18632/oncotarget.16438

**Published:** 2017-03-22

**Authors:** Jean-Francois Denis, K.E. Ludwig Scheckenbach, Anna Pfenniger, Merlijn J. Meens, Rob Krams, Lucile Miquerol, Steven Taffet, Marc Chanson, Mario Delmar, Brenda R. Kwak

**Affiliations:** ^1^ Department of Pathology and Immunology, University of Geneva, Geneva, Switzerland; ^2^ Department of Medical Specializations - Cardiology, University of Geneva, Geneva, Switzerland; ^3^ Department of Bioengineering, Imperial College, London, UK; ^4^ Aix-Marseille University, CNRS UMR 7288, Developmental Biology Institute of Marseille, Marseille, France; ^5^ Department of Microbiology, SUNY Upstate Medical University, Syracuse, NY, USA; ^6^ Departments of Pediatrics and of Cell Physiology and Metabolism, Geneva University Hospitals and University of Geneva, Geneva, Switzerland; ^7^ The Leon H. Charney Division of Cardiology, New York University School of Medicine, New York, NY, USA

**Keywords:** atherosclerosis, endothelium, Cx40, shear stress, IκBα

## Abstract

Connexins are proteins forming gap junction channels for intercellular communication. Connexin40 (Cx40) is highly expressed by endothelial cells (ECs) of healthy arteries but this expression is lost in ECs overlying atherosclerotic plaques. Low/oscillatory shear stress observed in bends and bifurcations of arteries is atherogenic partly through activation of the pro-inflammatory NFκB pathway in ECs. In this study, we investigated the relation between shear stress, Cx40 and NFκB. Shear stress-modifying casts were placed around carotid arteries of mice expressing eGFP under the Cx40 promoter (*Cx40^+/eGFP^*). We found that Cx40 expression is decreased in carotid regions of oscillatory shear stress but conserved in high and low laminar shear stress regions. These results were confirmed *in vitro*. Using phage display, we retrieved a binding motif for the intracellular regulatory Cx40 C-terminus (Cx40CT), *i.e*. HS[I, L, V][K, R]. One of the retrieved peptides (HSLRPEWRMPGP) showed a 58.3% homology with amino acids 5-to-16 of IκBα, a member of the protein complex inhibiting NFκB activation. Binding of IκBα (peptide) and Cx40 was confirmed by crosslinking and *en face* proximity ligation assay on carotid arteries. TNFα-induced nuclear translocation of NFκB in ECs was enhanced after reducing Cx40 with siRNA. Transfection of HeLa cells with either full-length Cx40 or Cx40CT demonstrated that Cx40CT was sufficient for inhibition of TNFα-induced NFκB phosphorylation. Finally, *Tie2Cre^Tg^Cx40^fl/fl^Apoe^-/-^* mice showed exaggerated shear stress-induced atherosclerosis and enhanced NFκB nuclear translocation. Our data show a novel functional IκBα-Cx40 interaction that may be relevant for the control of NFκB activation by shear stress in atherogenesis.

## INTRODUCTION

Atherosclerosis is a systemic lipid-driven inflammatory disease characterized by lesion formation in the intima of large and medium sized arteries. Rupture of atherosclerotic lesions is responsible for the majority of cardiovascular events such as myocardial infarction and stroke, which are the leading causes of death [1]. Atherosclerotic plaques display a variety of phenotypes: highly inflamed lesions with a large lipid pool and a thin fibrous cap are considered most vulnerable to rupture, whereas plaques with a high fibrous and smooth muscle cell content are generally recognized as more stable [2, 3]. Despite the fact that the entire arterial tree is exposed to systemic risk factors such as hypertension, hypercholesterolemia and diabetes, atherosclerotic plaques typically develop at geometrically predisposed areas, like the lesser curvature of bended vessel segments or near arterial branch points; *i.e*. sites that are exposed to low and/or oscillatory blood flow [4–6].

Endothelial cells (ECs) lining the inner surface of blood vessels are constantly exposed to wall shear stress, the frictional drag force created by blood flow. ECs respond to changes in shear stress by modulating intracellular signaling, which ultimately leads to alterations in gene expression and cell morphology [5]. Low and/or oscillatory shear stress in athero-susceptible regions triggers a dysfunctional endothelial phenotype with expression of pro-inflammatory and pro-thrombotic mediators and an impaired endothelial barrier function [4, 5].

Pro-inflammatory activation of ECs involves activation of the NFκB signaling pathway [7]. The NFκB transcription factor is normally sequestered within the cytoplasm by its inhibitor IκB, which upon stimulation is phosphorylated by IκB kinase (IKK) and directed for proteasomal degradation, thereby releasing NFκB which subsequently translocates to the nucleus where it initiates gene transcription. Indeed, *en face* staining and microarray studies revealed higher expression of NFκB/IκB pathway components in ECs exposed to disturbed flow. However, NFκB appeared only activated in a minority of the ECs and no significant differences were observed in expression of key adhesion molecules [8, 9]. This suggests that NFκB signal transduction is already primed for activation in disturbed flow regions of arteries on encountering an activation stimulus [10]. Using shear stress-modifying casts on mouse carotid arteries, Cuhlmann and colleagues demonstrated induction of NFκB activation and enhanced vascular adhesion molecule-1 (VCAM-1) expression at the low/oscillatory shear region downstream of the cast compared to the upstream low shear site [11]. Thus, low non-oscillatory and low oscillatory shear forces may have differential effects on EC activation and vascular inflammation.

The presence of gap junctions between ECs allows for a synchronized endothelial response by enabling the passage of ions and small metabolites between cells in contact [12]. Gap junction channels are made of connexins (Cx), a family of transmembrane proteins that consists of 21 members in human. Three connexins are expressed in arterial ECs, *i.e*. Cx37, Cx40 and Cx43 [12]. As they each form channels with different electrical properties and permeability, it is believed that they each play a unique role in endothelial homeostasis. We have previously shown that Cx40-mediated gap junctional communication contributes to maintenance of a quiescent non-activated endothelium by propagating adenosine-evoked anti-inflammatory signals between ECs [13]. Whether Cx40 participates to the formation of shear stress-induced communication compartments in the arterial endothelium, and thus to the functional separation of athero-prone and athero-protective regions, is however presently unknown.

## RESULTS

### Effect of shear stress on *in vivo* and *in vitro* Cx40 expression

**Figure 1:**
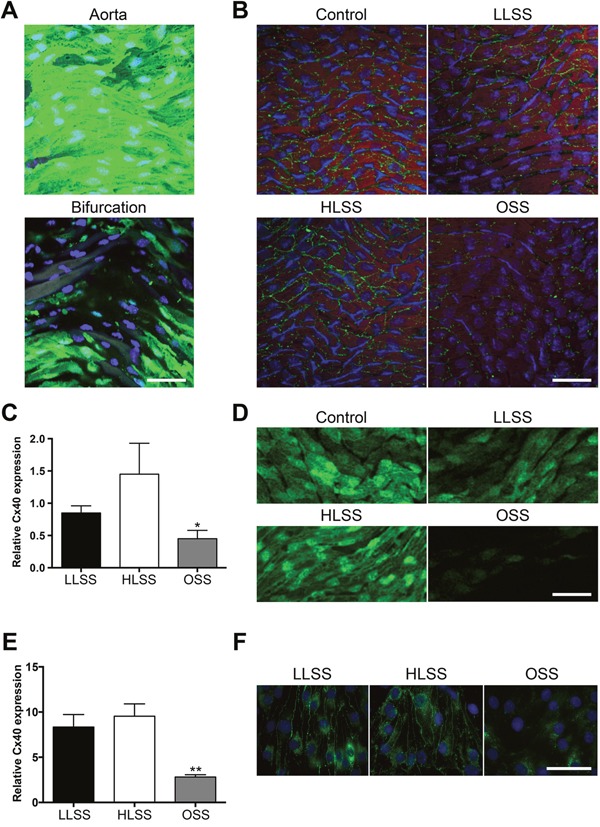
Expression of Cx40 is regulated by shear stress **(A)** Representative *en face* images of eGFP in longitudinally opened carotids of *Cx40^+/eGFP^* mice. eGFP (green) is highly expressed in the straight portions of the vessel (upper panel) but not at the iliac bifurcation (lower panel). DAPI (blue). **(B)** Cx40 expression (green) after modification of shear stress by a vascular cast in *Cx40^+/eGFP^* mice. Shown are the contralateral undisturbed vessel (control) and the regions upstream (LLSS), within (HLSS) and downstream of the cast (OSS). Evans Blue (red). DAPI (blue). **(C)** Quantification of (B); N=8. **(D)** eGFP after modification of shear stress by a vascular cast in *Cx40^+/eGFP^* mice. Shown are the contralateral undisturbed vessel (control) and the regions upstream (LLSS), within (HLSS) and downstream of the cast (OSS). **(E)** Cx40 expression in bEnd.3 cells exposed to static, HLSS, LLSS, OSS conditions for 24 hours was assessed by real-time qPCR. N=5. **(F)** Representative images of Cx40 expression (green) in bEnd.3 cells exposed to HLSS, LLSS, OSS for 24 hours. Scale bar represents 50 μm for in (A), (B) and (D), and 40 μm in (F).

To investigate possible effects of shear stress on endothelial Cx40 expression, we first performed *en face* immunofluorescence on straight parts of the abdominal aorta and on the iliac bifurcation of *Cx40^+/eGFP^* mice that express eGFP controlled by the Cx40 promoter. Whereas eGFP was highly present in the straight portions of the aorta that are exposed to physiological high laminar shear stress (HLSS), it was considerably reduced at the level of the iliac bifurcation, where an oscillatory shear stress (OSS) is anticipated (Figure 1A). Remarkably, the expression of eGFP varied considerably in the iliac bifurcation region and it was also not uniform in the abdominal aorta where cells expressing high levels of eGFP could be found adjacent to cells with a much lower eGFP expression. Then, shear stress-modifying casts were placed around the right common carotid artery of *Cx40^+/eGFP^* mice as previously published [14, 15]. First, Cx40 expression was determined in the casted and in the contralateral control carotids by *en face* immunofluorescence at 1 week after cast placement. As shown in Figure 1B, Cx40 was highly expressed in undisturbed control carotid arteries. Whereas this high expression was maintained in the laminar shear stress regions located inside the cast (HLSS) and upstream of the cast (low laminar shear stress; LLSS), Cx40 was decreased in the OSS region downstream of the cast. Quantification of immunosignal for Cx40 in 8 mice showed a reduction to more than half in the region subjected to OSS (Figure 1C). These results were confirmed by analyses of the eGFP signal in casted *Cx40^+/eGFP^* mice (Figure 1D). Moreover, visualization of the eGFP signal in casted carotids showed a similar non-uniformity in expression levels of adjacent cells as observed at the level of the abdominal aorta. To exclude influence of systemic factors and circulating cells, flow-dependent Cx40 regulation was also investigated *in vitro*. The mouse EC line bEnd.3 that constitutively expresses all vascular connexins [16] and is known to respond to changes in shear stress [15], was exposed to HLSS (20 dynes/cm^2^), LLSS (5 dynes/cm^2^) or OSS (5 dynes/cm^2^; 1Hz) for 24 hours or kept under static conditions. Again, OSS reduced Cx40 transcripts and protein when compared to HLSS as well as LLSS, as shown by quantitative PCR (qPCR) and immunofluorescence, respectively (Figure 1E and 1F). Thus, Cx40 expression in ECs is modulated by blood flow, being decreased in regions exposed to OSS.

### Phage display to identify potential binding partners

Cx40 is traditionally considered a gap junction protein essential for direct cell-to-cell communication in the vasculature [12]. However, recently increasing attention is given to channel-independent functions of connexins [17, 18]. Therefore, we carried out a high-throughput phage display screening in search of peptidic sequences that bind to the intracellular C-terminus of Cx40 (Cx40CT). We analyzed the sequence of the insert retrieved from a total of 118 plaques. Of the estimated 2.5×10^9^ different sequences presented in the phage display, 22 were captured by recombinant Cx40CT. Table 1 shows the corresponding peptide sequences and the number of plaques analyzed that contained the same sequence. When compared to the pre-bound library, basic residues were more frequently found in the captured peptides, whereas acidic residues showed minimal differences (Table 2). Strikingly, half of the captured peptide sequences presented amino acids HS[I, L, V][K, R] as part of their sequence (Table 1). We calculated the probability of occurrence of this motif by chance alone taking into account the abundance of individual amino acids in the library prior to screening. The actual occurrence of this specific sequence appeared more than 300 times higher than its expected probability (Table 3). As Cx43CT and Cx37CT are known for their preferential capture of RXP and RXXP motifs, we also calculated the actual occurrence for these motifs in our Cx40CT phage display. We found a slight (1.8 times) increased occurrence of the RXP motif whereas the RXXP motif was even less frequently encountered than its expected probability (Table 3). Altogether, the data support the notion that Cx40CT binds with enhanced selectivity to peptides containing the motif HS[I, L, V][K, R].

**Table 1:** Alignments of peptidic sequences derived from the phage display screen

15	5’-**HSLR****PEW****RMP****GP**-3’ *
1	5’-**TM****HSLR****PEW****RMP**-3’
2	5’-**HSLK****P**S**W**LLL**G**Y-3’
1	5’-**HSVK****P**D**W**AQMLR-3’
34	5’-**HSVK****P**VVNLILR-3’
2	5’-**HSLK****P**SLKQLAI-3’
10	5’-**HSIR**TY**W**QSAQ**P**-3’
2	5’-**HSLR**ED**W**TLRMQ-3’
1	5’-**HSVK**HDF**R**LLTK-3’
19	5’-**HSIR**LHTYPHMK-3’
1	5’-**HSIR**SSHLHMFT-3’
18	5’-Y**SLR**ADS**R**W**MP**S-3’
1	5’-SG**H**Q**L**-LLN**KMP**N-3’
1	5’-APRLPQ**SL**L**P**QL-3’
1	5’-S**H**A**L**PLT**W**STAA-3’
2	5’-APPGN**WR**NYL**MP**-3’
1	5’-AP**P**MS**R**QSFDGV-3’
1	5’-NFME**SL**PRLGMH-3’
1	5’-LLADTT**H**-H**RP**WT-3’
1	5’-MEGQYKSNLLFT-3’
1	5’-NTELTSYGPPPA-3’
2	5’-TMGFTAPRFPHY-3’

* IκBα-like

Red: HS[I, L, V][K, R] motif; Blue: [K, R]XP motif; Green: [K, R]XXP motif

Numbers represent occurrence of the peptidic sequence in the phage display.

**Table 2:** Frequency of acidic and basic residues in phage display sequences

	Cx40 display	library
Asp	2.3%	2.8%
Glu	2.3%	3.1%
His	8.3%	6.3%
Lys	3.8%	2.8%
Arg	7.2%	4.7%

**Table 3:** Frequency of HS[I, L, V], [K, R] or [K, R]XP or [K, R]XXP motifs in the Cx40CT phage display versus theoretical occurrence

	HS[I, L, V], [K, R]	[K, R]XP	[K, R]XXP
Theoretical frequency	0.157%	12.54%	11.3%
Experimental frequency	50.0%	22.7%	4.5%
Ratio Exp/Th	318.9	1.8	0.4

Subsequent sequence alignments using the Basic Local Alignment Search Tool (BLAST) against the National Center for Biotechnology Information protein database indicated homology between two of the retrieved sequences and the N-terminus (NT) of IκBα, a member of the protein family that inhibits the nuclear translocation of NFκB. The most frequently retrieved IκBα-like sequence (i.e. HSLRPEWRMPGP indicated with an asterisk in Table 1) contained the HS[I, L, V][K, R] motif and showed a 58.3% homology with amino acids 5 to 16 of IκBα. Given the biological importance of NFκB translocation in arterial ECs in regions exposed to OSS and the specific decrease of Cx40 in this region, we continued with further characterization of interactions between this peptide and Cx40CT.

### *In vitro* and *in situ* binding of Cx40 and IκBα

**Figure 2:**
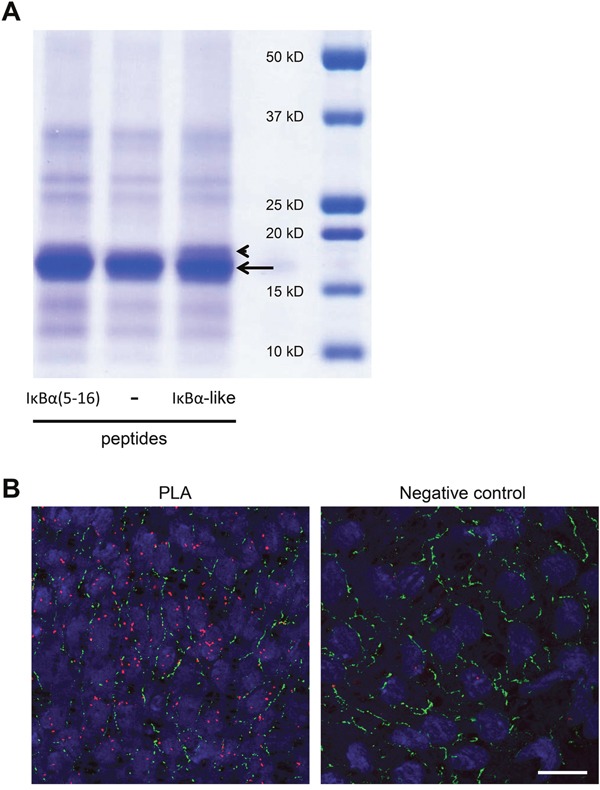
Cx40 and IκBα interact *in vitro* and *in vivo* **(A)** Cross-linking experiment with Cx40CT and peptides. Binding of IκBα-like or IκBα(5-16) peptides to Cx40CT was assessed after incubation with the chemical cross-linker BS^3^. All lanes show a band at ~16-17 kDa representing Cx40CT (arrow). The first and third lanes of the panel show an additional band at ~17-18kDa (arrow head), which is absent in lane 2, where the peptide is missing. **(B)** Representative *en face* images of PLA (out of 3 experiments) performed with antibodies targeting Cx40 and IκBα on rat carotid endothelium. Close proximity of Cx40 and IκBα (red) is observed in the intracellular compartment as well as at cell-cell contacts (left panel). Control assays revealed that the red staining observed was no longer observed after omitting either the Cx40 or the IκBα antibody from the PLA (right panel). Cx37 staining (green) is used to highlight the intercellular gap junctions. DAPI (blue). Scale bar represents 20 μm.

We used cross-linking experiments with BS^3^ to confirm the intermolecular interaction between Cx40CT and the peptide showing the highest homology to IκBα (called: “IκBα-like”) as well as a 12-mer peptide corresponding to sequence 5-16 of IκBα (called: “IκBα(5-16)”). As indicated by the arrow in Figure 2A, a band at ~16-17 kDa representing Cx40CT was seen in all samples, however in the first and the third lane, where samples contained IκBα(5-16) or IκBα-like peptides respectively, a supplementary band, corresponding to the Cx40CT-peptide complex, was seen just above the Cx40CT band (as indicated by the arrowhead). Control experiments in the absence of peptides (Figure 2A, lane 2) or in the absence of BS^3^ (not shown) did not reveal this supplementary band. We further investigated the potential interaction between Cx40 and IκBα using *in situ* proximity ligation assays (PLA). This method employs proximity probes – oligonucleotides attached to secondary antibodies – to guide formation of circular DNA strands when bound in close proximity (~30 nm). The DNA circles subsequently serve as templates for localized rolling-circle amplification, allowing visualization of protein-protein interactions [19]. As shown in Figure 2B (left panel), Cx40 and IκBα were detected in close proximity in carotid endothelium (red signal) most frequently as an intracellular signal but sometimes also at cell-cell interfaces that were highlighted by a green Cx37 immunosignal. No (red) signal was detected in negative controls from which the antibody detecting IκBα was omitted (Figure 2B, right panel). Altogether, these data show that Cx40 and IκBα are interacting proteins and that this interaction is most apparent in the intracellular compartment.

### Functional effect of Cx40-IκBα interaction

**Figure 3:**
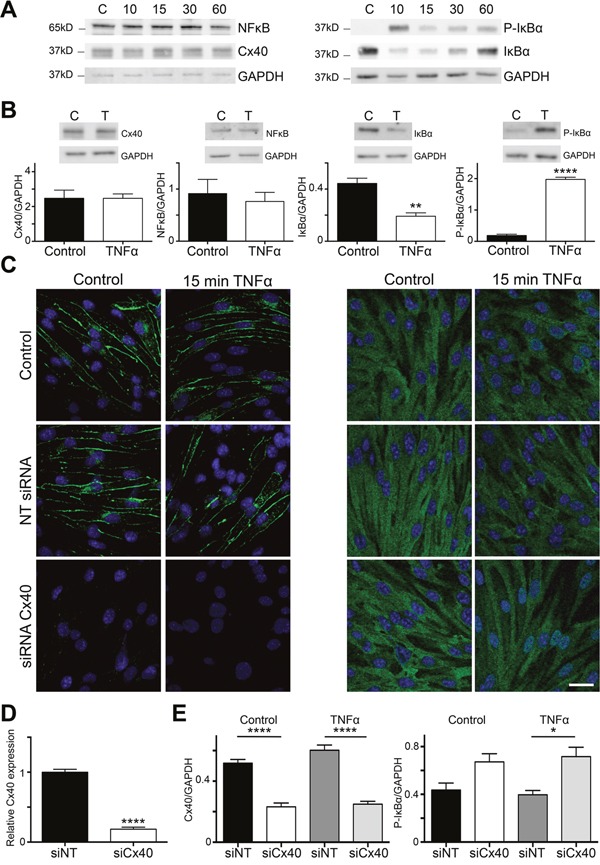
Cx40 dampens NFκB nuclear translocation in a mouse EC line **(A)** Lysates of bEnd.3 cells incubated or not with 10 ng/ml TNFα for 10, 15, 30 or 60 min were immunoblotted against NFκB, Cx40, IκBα, Phospho-IκBα or GAPDH. Whereas expression levels of NFκB, Cx40, and GAPDH were not affected by short-term stimulation with TNFα, the treatment induced phosphorylation and degradation of IκBα. **(B)** Quantification of (A) under control conditions or after incubation with 10 ng/ml TNFα for 15 min. N=3. **(C)** Expression of Cx40 (left panels) or NFκB (right panels; both in green) in control bEnd.3 cells and after 15 min stimulation with 10 ng/ml TNFα treated or not with siRNA for Cx40 or NT-siRNA. Note that in ECs in which Cx40 was silenced with siRNA (lower panels), NFκB translocation to the nucleus was enhanced after stimulation with TNFα. DAPI (blue). Scale bar represents 15 μm. **(D)** Cx40 expression in bEnd.3 cells exposed to Cx40 siRNA or NT-siRNA was assessed by real-time qPCR. N=3. **(E)** Expression of Cx40 (left) or Phospho-IκBα (right) in control bEnd.3 cells and after 15 min stimulation with 10 ng/ml TNFα treated with siRNA for Cx40 or NT-siRNA. N=3.

To study possible functional effects of the interaction between Cx40 and IκBα, we used bEnd.3 cells stimulated with 10 ng/ml TNFα for various periods (0-60 min). As shown in Figure 3A and 3B, expression levels of Cx40 and NFκB were not affected by TNFα stimulation for periods up to 60 min. Stimulating bEnd.3 cells for 10 min with TNFα induced IκBα phosphorylation at positions 32 and 36 (Figure 3A), an event that is known to precede phosphorylation of NFκB and its subsequent translocation to the nucleus [7]. As expected, phosphorylation of IκBα was accompanied by a concomitant degradation of the protein, visible in the Western blot as a decreased signal at 10 and 15 min (Figure 3A), after which the protein was re-expressed at 60 min. Phosphorylation of IκBα was increased by 8-fold and the IκBα protein was reduced by half after stimulating bEnd.3 cells for 15 min with TNFα (Figure 3B).

Next, we exposed bEnd.3 cells to Cx40 siRNA and studied NFκB translocation by immunofluorescence. Cx40 expression was virtually absent after exposure to 25 nM Cx40 siRNA, whereas the same concentration of non-targeting siRNA (NT siRNA) did not affect its expression, both under control conditions or after 15 min TNFα stimulation (Figures 3C, left panels, and 3D). Exposure to TNFα did not induce NFκB translocation to the nucleus in control or NT siRNA-transfected bEnd.3 cells (Figure 3C, upper and middle right panels). Interestingly, substantial TNFα-induced nuclear translocation of NFκB was observed in bEnd.3 cells after effective inhibition of Cx40 by siRNA (Figure 3C, bottom right panel). Moreover, we also observed a significant increase in IκBα phosphorylation after 15 min stimulation with TNFα in bEnd.3 cells after effective inhibition of Cx40 by siRNA (Figure 3E). Overall, the data suggest that the interaction of IκBα with Cx40 decreases its phosphorylation, thereby resulting in reduced nuclear translocation of NFκB.

### Functional effect of the Cx40-IκBα interaction

**Figure 4:**
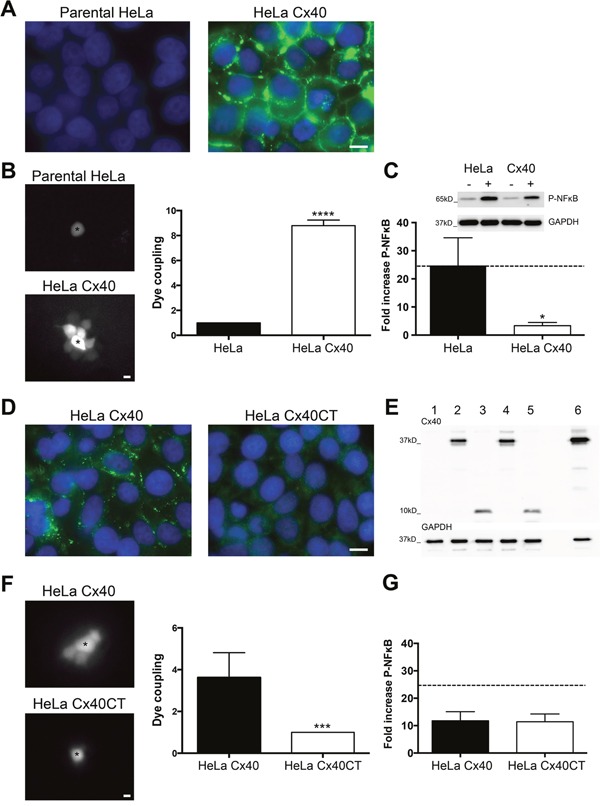
Cx40CT is sufficient for inhibition of TNFα-induced NFκB activation **(A)** Cx40 immunostaining (green) in communication-incompetent HeLa cells (left panel) and in HeLa cells stably transfected with Cx40 (right panel). DAPI (blue). **(B)** Intercellular communication was measured by Lucifer Yellow microinjection during 3 min. Images are representative examples of Lucifer Yellow diffusion in parental HeLa cells (upper panel, N=6) and in HeLa cells stably transfected with Cx40 (lower panel, N=10). Asterisks indicate the microinjected cells. **(C)** Western blots (upper panel) showing the induction of Phospho-NFκB after 5 min stimulation with 20 ng/ml TNFα (+) as compared to control conditions (-) in parental HeLa cells and in HeLa cells stably transfected with Cx40. Expression of Cx40 reduced TNFα-induced NFκB phosphorylation (lower panel). N=3. **(D)** Cx40 immunostaining (green) in communication-incompetent HeLa cells transiently transfected with full-length Cx40 (left panel) or with Cx40CT (right panel). DAPI (blue). **(E)** Lysates of parental HeLa cells (lane 1) or transiently transfected with Cx40 (lanes 2 and 4) or Cx40CT (lanes 3 and 5) or stably transfected with Cx40 (lane 6) were immunoblotted against Cx40 and GAPDH. **(F)** Intercellular communication was measured by Lucifer Yellow microinjection during 5 min. Images are representative examples of Lucifer Yellow diffusion in HeLa cells transiently transfected with Cx40 (upper panel, N=8) or Cx40CT (lower panel, N=6). Asterisks indicate the microinjected cells. **(G)** Induction of Phospho-NFκB after 5 min stimulation with 20 ng/ml TNFα in HeLa cells transiently transfected with Cx40 or Cx40CT. Expression of Cx40 or Cx40CT revealed a similar protection against TNFα-induced NFκB phosphorylation. N=3. Scale bar represents 10 μm in (A) and (D), and 15 μm in (B) and (F).

To investigate whether the inhibitory effect of Cx40 on NFκB translocation in ECs could be generalized to other cell types, we used communication-deficient parental HeLa cells and HeLa cells stably transfected with Cx40. Immunofluorescence confirmed absence of Cx40 in the parental cells and showed a high level of Cx40 expression in stably transfected HeLa cells (Figure 4A). Moreover, microinjection of Lucifer Yellow in one Cx40-transfected cell resulted in its spread to ~8 neighboring cells. In contrast, no cell-to-cell coupling was observed between parental HeLa cells (Figure 4B). Finally, we detected the induction of Phospho-NFκB by 20 ng/ml TNFα in parental and stably Cx40-transfected HeLa cells (Figure 4C). Whereas 5 min TNFα exposure induced a 25-fold increase in NFκB phosphorylation in parental cells (Figure 4C, black bar and dotted line), the induction of NFκB phosphorylation was only 3-fold in Cx40-transfected HeLa cells (Figure 4C, white bar).

To study whether the inhibitory effect of Cx40 on NFκB translocation was dependent on functional gap junction channels or could be ascribed to channel-independent effects, we transiently transfected communication-deficient HeLa cells using a pIRES-eGFP plasmid containing full-length Cx40 or Cx40CT. As expected, immunostaining revealed full-length Cx40 at cell-cell interfaces, whereas Cx40CT was detected only intracellularly in transfected HeLa cells (Figure 4D). Expression of Cx40 or Cx40CT was confirmed by Western blotting and demonstrated no major differences in expression levels between both proteins (Figure 4E). Moreover, Lucifer Yellow microinjection in one transiently Cx40-transfected cell resulted in its spread to ~4 neighboring cells, whereas no cell-to-cell coupling was observed between HeLa cells transiently transfected with Cx40CT (Figure 4F). Finally, 5 min TNFα exposure induced about 12-fold increase in NFκB phosphorylation in both Cx40 or Cx40CT transiently transfected HeLa cells (Figure 4G). Thus, the inhibitory effect of Cx40 on NFκB phosphorylation/activation is not restricted to ECs and is independent of functional gap junction channels.

### Exaggerated induction of atherosclerosis in EC-Cx40-deficient mice

**Figure 5:**
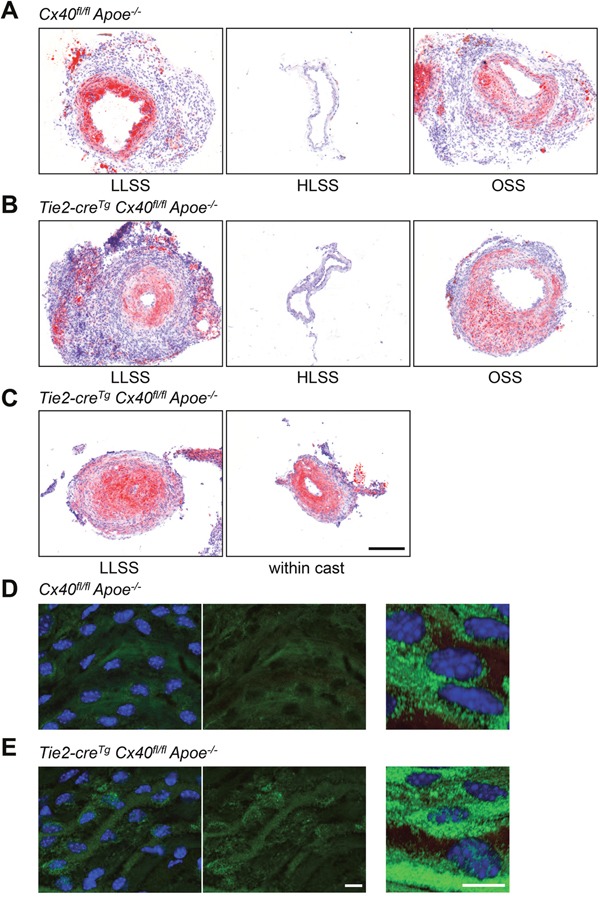
Exaggerated flow-induced atherosclerosis in *Apoe^-/-^* mice with endothelial deletion of Cx40 Representative images of SudanIV stainings are shown for the 3 flow regions of casted vessels (LLSS, HLSS, OSS) from *Cx40^fl/fl^Apoe^-/-^* **(A)** and *Tie2-cre^Tg^Cx40^fl/fl^Apoe^-/-^* **(B, C)** mice after 6 weeks of high-cholesterol diet. Intimal thickening was present in regions subjected to LLSS and OSS in *Cx40^fl/fl^Apoe^-/-^* mice (A). Intimal thickening in response to LLSS and OSS was increased in *Tie2-cre^Tg^Cx40^fl/fl^Apoe^-/-^* mice (B). The increased atherosclerotic response caused in half of the *Tie2-cre^Tg^ Cx40^fl/fl^Apoe^-/-^* mice a complete occlusion of the LLSS area that gave rise to atherosclerotic lesions within the cast (C). N=6-8 animals per group. Scale bar = 200 μm. **(D, E)** *En face* immunostaining for NFκB (in green) in carotid arteries of *Cx40^fl/fl^Apoe^-/-^* (D) and *Tie2-cre^Tg^Cx40^fl/fl^Apoe^-/-^* (E) mice. Note that NFκB signal was mostly cytoplasmic in *Cx40^fl/fl^Apoe^-/-^* mice and more frequently localized to the nucleus in *Tie2-cre^Tg^Cx40^fl/fl^Apoe^-/-^* mice. DAPI (blue). Scale bar represents 10 μm.

Whereas low shear stress levels prime ECs by increasing NFκB expression, OSS seems to induce arterial inflammation by promoting not only NFκB expression but also its nuclear localization in ECs [11]. To investigate the role of endothelial Cx40 in flow-induced atherosclerosis *in vivo*, we used atherosclerosis-susceptible mice in which Cx40 was deleted from the endothelium [13]. Shear stress-modifying casts were placed around the right common carotid artery and the mice were fed a high-cholesterol diet for 6 weeks. As shown previously for *Apoe^-/-^* mice [14, 20], *Cx40^fl/fl^Apoe^-/-^* controls showed intimal thickening in regions exposed to LLSS or OSS (Figure 5A). As expected, flow-induced atherosclerotic remodeling was significantly increased in *Tie2Cre^Tg^Cx40^fl/fl^Apoe^-/-^* carotids (Figure 5B and 5C) with 0.97±0.01 of the vessel surface being occluded in the LLSS region of *Tie2Cre^Tg^Cx40^fl/fl^Apoe^-/-^* carotids *vs*. only 0.54±0.06 in carotids of *Cx40^fl/fl^Apoe^-/-^* mice. Of note, the increased atherosclerotic response resulted in complete occlusion of the LLSS area, which, in turn, gave rise to atherosclerotic lesions within the cast in half of the *Tie2-cre^Tg^Cx40^fl/fl^Apoe^-/-^* mice (see Figure 5C for an example). We further investigated the effect of endothelial deletion of Cx40 on the *in situ* location of NFκB by *en face* immunostaining for NFκB in longitudinally opened carotids of *Tie2Cre^Tg^Cx40^fl/fl^Apoe^-/-^* and *Cx40^fl/fl^Apoe^-/-^* mice. As shown in Figure 5D, NFκB was detected mostly as a cytoplasmic signal in carotid arteries of *Cx40^fl/fl^Apoe^-/-^* control mice. However, NFκB signal frequently co-localized with nuclei in carotids of *Tie2Cre^Tg^Cx40^fl/fl^Apoe^-/-^* mice (Figure 5E). Overall, our study demonstrates that Cx40 and IκBα are interacting proteins. Moreover, absence of Cx40 from the endothelium enhances NFkB nuclear translocation and exaggerates induction of atherosclerosis both in regions of OSS and LLSS.

## DISCUSSION

During early atherogenesis, leukocytes are recruited to the arterial intima through secretion of chemokines and expression of adhesion molecules such as VCAM-1, intercellular adhesion molecule-1 (ICAM-1) and P-selectin by activated ECs. A key regulator of these pro-inflammatory processes is the transcription factor NFκB [7]. In a quiescent endothelium, the NFκB dimer is kept in an inactive conformation through binding to the inhibitory IκB protein. A variety of pro-inflammatory signals, like TNFα or OSS, trigger the activation of the IKK complex resulting in the phosphorylation of IκBα at amino acids 32 and 36. Subsequent proteosomal degradation of IκBα releases the NFκB dimer (mostly p50-RelA) for nuclear translocation and initiation of gene transcription. NFκB signaling is subjected to a number of regulatory mechanisms, *e.g*. at the level of its inhibitor IκBα. Importantly, NFκB activation rapidly induces IκBα expression, thus providing an auto-regulatory negative feedback loop [21]. In addition, IκBα stability is controlled by the COP9 signalosome that regulates deneddylation and deubiquitination processes, even under NFκB activating conditions [22, 23]. In this study, we add Cx40 to the collection of proteins that critically regulate endothelial NFκB signaling.

ECs display a large degree of heterogeneity. *I.e*. ECs at athero-prone sites express relatively high levels of NFκB, whereas ECs at athero-protected sites express low amounts of NFκB proteins and are resistant to activation [10]. This spatial difference in EC behavior has been attributed to spatial variation in hemodynamic forces: athero-protected sites are exposed to high rates of unidirectional flow, while athero-susceptible sites experience lower rates of unidirectional or oscillatory flow [5]. Using shear stress-modifying casts, Cuhlmann and colleagues elegantly demonstrated that low shear may prime ECs for inflammatory activation by inducing NFκB expression, whereby oscillatory shear promoted both expression and nuclear localization (activation) of NFκB [11]. The reason for this difference was not investigated. Here, we observed a reduction in Cx40 in response to OSS, whereas Cx40 expression was preserved at sites of LLSS. We further demonstrated that inhibition of Cx40 expression favors the translocation of NFκB in ECs *in vitro* (Figure 3C) as well as *in situ* (Figure 5D and 5E). Finally, the endothelium in aortic sinuses (a typical OSS location) of 10 weeks-old *Tie2Cre^Tg^Cx40^fl/fl^Apoe^-/-^* mice expresses the NFκB-inducible protein VCAM-1 while this protein is absent at this location in young control mice [13]. Together, these results suggest that the presence of Cx40 in the endothelium of LLSS regions dampens NFκB activation despite increased expression levels in these regions.

Gap junction-mediated intercellular communication (GJIC) governed by Cx37, Cx40 and Cx43 between arterial ECs is important in vascular physiology as it synchronizes the response of ECs to various agonists [12]. For instance, GJIC assures a homogeneous increase in cytosolic calcium and subsequent activation of eNOS following exposure to histamine despite a focal expression pattern of the histamine receptor H1 in arterial ECs [24]. Moreover, connexins affect vascular pathology, including atherosclerosis. Whereas Cx43 plays an atherogenic role [25, 26] Cx37 and Cx40 are athero-protective [13, 27]. Interestingly, the expression of the three connexins seems differently regulated by arterial shear stress patterns; Cx43 is abundantly expressed in aortic endothelia localized downstream of ostia of branching vessels and at flow dividers [28]. Moreover, HLSS enhances Cx37 expression in carotid ECs by inducing its transcription factor KLF2, which increases GJIC and contributes to EC synchronization [15]. In contrast to Cx40, Cx37 expression is decreased in OSS as well as LLSS regions [15]. Finally, shear stress-dependent endothelial Cx40 expression in small arteries and arterioles plays an important role in signal propagation along blood vessel walls, thereby modulating vessel diameter and organ blood flow [29]. Using a mouse model expressing eGFP under the control of the Cx40 promoter, our study reveals for the first time heterogenous Cx40 promoter activity in ECs of large arteries, which is most pronounced in areas of LLSS but also present in HLSS regions. This non-uniform Cx40 expression in adjacent ECs led us to search for GJIC-independent functions of Cx40 and this led to the identification of IκBα as a new binding partner for the intracellular Cx40CT. The IκBα-like peptide contains the HS[I, L, V][K, R] motif as part of its sequence. The occurrence of this specific motif in our Cx40CT phage display was more than 300 times higher than its expected probability. Moreover, this specific motif was not found in earlier phage displays for Cx43CT or Cx37CT [30, 31], suggesting that IκBα may be an exclusive binding partner for Cx40 in ECs. Our subsequent transfection studies with full-length Cx40 and Cx40CT revealed that the inhibitory effect of Cx40 on IκBα was not restricted to ECs and was independent of functional gap junction channels. Further studies should aim to identify the binding site of IκBα-like in the Cx40CT as this may represent a basis for the development of novel pharmacophores inhibiting pro-inflammatory NFκB signaling in multiple cell types and diseases.

In summary, our results show that Cx40CT reduces endothelial activation by impairing NFκB activation. Thus, Cx40 might confer athero-protection in the arterial endothelium by controlling inflammatory responses. As Cx40 is reduced by OSS, this mechanism may contribute to the increased atherosclerotic plaque formation at predilection sites where OSS is predominant, such as arterial bifurcations.

## MATERIALS AND METHODS

### Animals

Animal experimentation conformed to the *Guide for the Care and Use of Laboratory Animals* published by the US National Institutes of Health and was approved by Swiss cantonal veterinary authorities. The generation of heterozygous mice in which eGFP expression is controlled by the *Cx40* promoter (*Cx40^eGFP/+^* mice) has been described elsewhere in detail [32]. Atherosclerosis-susceptible mice with endothelial deletion of Cx40, *i.e. Tie2Cre^Tg^Cx40^fl/fl^Apoe^-/-^* mice and *Cx40^fl/fl^Apoe^-/-^* controls, on a C57BL/6 background, have been generated as previously described [13, 33]. Genotypes of the different mice were verified by PCR using previously described protocols [13, 32, 33]. All mice were kept in conventional housing.

### *In vivo* alteration of shear stress

A shear stress-modifying cast was used to induce defined changes in shear stress, as previously described [15, 34]. In brief, mice were anesthetized with 5% isoflurane inhalation for induction, followed by 2% isoflurane for maintenance of anesthesia. The anterior cervical triangle was accessed by a sagittal anterior neck incision. Both halves of the vascular cast were placed around the right common carotid artery and fixed with a suture. Post-operative analgesia was performed with i.p. Buprenorphinum (0.05 mg/kg) injections for 3 days. *Cx40^eGFP/+^* mice were fed a normal chow diet and were sacrificed 1 week after cast placement following general anesthesia with ketamine 100 mg/kg and xylasine 10 mg/kg i.p. Carotids were retrieved, opened longitudinally, pinned in silicone dishes and were further processed for immunofluorescence. Immunosignal in the casted carotid regions were normalized to the signal in the undisturbed contralateral control carotid artery. *Tie2Cre^Tg^Cx40^fl/fl^Apoe^-/-^* mice and *Cx40^fl/fl^Apoe^-/-^* controls were fed a high-cholesterol diet for 6 weeks. After perfusion with 0.9% NaCl, casted vessels and contralateral undisturbed control vessels were excised and the cast was removed. Samples were embedded in OCT compound and snap frozen. Five μm-thick serial cryosections were obtained from the 3 flow regions determined by the conical, progressively constrictive shape of the cast (upstream – LLSS, inside – HLSS, downstream – OSS) and from the control vessel. Sections were stained with general histochemical methods such as Hematoxylin/Eosin, picrosirius red (for collagen) and Sudan IV (for lipids).

### Phage display

We used phage display to identify potential ligates to the C-terminal domain of Cx40 (Cx40CT). The protocol was similar to that previously described [30, 31]. Briefly, recombinant Cx40CT (amino acids 231-358) was used as bait. A well of a 24-well plate was coated with 15 μg of recombinant Cx40CT. The well was subsequently treated with blocking buffer (0.1 M NaHCO_3_ (pH 8.6), 5 mg/mL BSA, 0.02% NaN_3_) for 1 hour to prevent non-specific binding. A phage library consisting of 2.7×10^9^ different M13KE bacteriophage displaying a random 12-mer peptide in their minor coat protein (Ph.D.-12™ Phage display peptide library kit; New England BioLabs Inc.) was first pre-cleared in an uncoated well, and then presented to the bait protein. Low-affinity binders were first eluted using a 100 μg/ml solution of free Cx40CT in TBS. The well was overlaid with a culture of *E. coli* ER2738, which was then amplified for 4.5 hours. The amplified phage were precipitated with PEG-NaCl. The extracted phage were subsequently used for a new round of panning. After four rounds, the phage were grown on a lawn of *E. coli* ER2738 for plaque purification. A total of 118 plaques, each one representing a single clone, were amplified for 4.5 hours. Phage were precipitated with PEG/NaCl and their ssDNAs were extracted and sequenced. The sequences were analyzed using the ExPasy translate tool. Homology to human proteins was determined using the NCBI protein-protein BLAST (Basic Local Alignment Search Tool).

### Motif analysis

The significance of particular motifs in the retrieved phage display sequences was assessed by comparing their actual frequency to their theoretical occurrence based on the experimental ratio of each amino acid. If for example the frequency of K in the display sequences is 3.8%, the frequency of R is 7.2% and the frequency of P is 11.4%, then the probability of finding a [K, R]XP motif in a 12-mer would be p([K, R]XP) = 10 x (p(K) + p(R)) x p(P) = 12.4% even without a selection for this motif. These probabilities were then compared to the obtained data.

### Cross-linking experiments and proximity ligation assays

For cross-linking, recombinant Cx40CT (0.25 mM) and a peptide (0.5 mM) were incubated for 1 hour at room temperature (RT) with 1 mM of the cross-linker reagent BS^3^ at pH 7.45. The reaction was subsequently blocked with 100 mM ethanolamine for 10 min. The samples were separated by SDS-PAGE (4-20%) and stained with Coomassie-Blue.

Proximity ligation assays (PLA) were performed using the DUOLink™ kit (Olink) according to the manufacturer's protocol. In brief, rat carotid arteries were opened longitudinally, pinned out on silicone dishes, fixed with 100% methanol for 5 min at -20°C, permeabilized with 0.2% Triton X-100 in PBS for 1 hour, charges neutralized with 0.5 M NH_4_Cl in PBS for 15 min and blocked in 2% bovine serum albumin (BSA) for 30 min. Subsequently, primary antibodies against Cx40 (Cx40-A; Alpha-Diagnostics lot #175455A8.6; 1/200) and IκBα (L35A5; Cell Signaling; 1/100) in blocking solution were applied overnight at 4°C. Next, secondary PLA probes (PLUS and MINUS) in blocking solution were applied and proximity ligation was performed using the Duolink detection reagent RED kit according to the manufacturer's protocol. Cell-cell junctions were counterstained with Cx37 (Cx37A11-A; Alpha-Diagnostics, lot #175859A5-L; 1/50) diluted in 2% BSA and nuclei were stained with DAPI. Finally, the sections were mounted using Vectashield (Vector Laboratories) and analysed using a LSM510-Meta confocal microscope (Zeiss).

### Cell culture and transfection

Different cell lines were used: a) a mouse endothelial cell line (bEnd.3) which endogenously expresses all three endothelial connexins [16], b) a communication-incompetent sub-clone of human HeLa cells (American Type Culture Collection) and c) the afore-mentioned HeLa cells stably transfected with murine Cx40 [35]. Cells were grown in DMEM (41966-029, Gibco) supplemented with 10% fetal bovine serum, 5000 U/l penicillin and 5 mg/ml streptomycin (Mediatech). The medium of the stably Cx40-transfected HeLa cells was supplemented with 0.5 μg/ml puromycin (Sigma). bEnd.3 cells were grown on dishes or coverslips coated with 1.5% gelatin. For flow experiments, cells were counted and grown in gelatin-coated u-slides VI^0.4^ (Ibidi). Immunosignals in LLSS, HLSS and OSS were normalized to the signal in static conditions.

Silencing Cx40 in bEnd.3 cells using short interfering RNA (siRNA) was performed using SMARTpool ON-TARGETplus Gja5 siRNA (Dharmacon) specific for mouse Cx40 (mCx40). The ON-TARGETplus Non-targetting Pool (Dharmacon), which does not target known mouse sequences, was used as negative control. Transfections were performed under serum-free conditions, with 25 nM siRNA and 2 mg/l DharmaFECT4. Efficient Cx40 silencing was determined by qPCR and immunofluorescence.

Transient transfections with pIRES2-eGFP containing cDNA coding for murine Cx40 or murine Cx40CT [36] were performed using Lipofectamine LTX&PLUS™ Reagent (Thermofisher) according to the manufacturer's instructions. Briefly, 1×10^6^ communication-incompetent HeLa cells were grown for 24 hours in 6-well plates. For transfection, 2.5 μg of plasmid, 2.5 μl of PLUS Reagent and 12.5 μl of Lipofectamine LTX were mixed in 300 μl Opti-MEM Medium (Gibco) and incubated at RT for 5 min. Then, the plasmid-Lipofectamine LTX complexes were added to each well supplemented with 1.7 ml DMEM and incubated for 24 hours at 37°C. Transfected cells were selected with 500 μg/ml G418 (Gibco) added to the complete medium (changed every 2 days). Purity of the transfectants was followed every passage by FACS (BD Accuri C6). Experiments were performed when 70-80% of the cells were eGFP positive.

### Quantitative PCR

Total RNA from bEnd.3 cells was obtained using the NucleoSpin kit (Macherey-Naegel). Reverse transcription was performed using the Quantitect Reverse Transcription kit (Qiagen) and quantitative PCR was performed with the ABI Prism StepOnePlus Sequence Detection System (Applied Biosystems) using the TaqMan Fast Universal master mix (Applied Biosystem). Mouse Cx40 (Mm00433619_S1; Thermofisher Scientific) and GAPDH (Mm99999915_G1; Thermofisher Scientific) were used as Taqman probes. Cx40 expression was normalized to GAPDH expression.

### Immunofluorescence microscopy

Cx40 and NFκB immunofluorescent staining on carotid arteries, HeLa cells and bEnd.3 cells was performed as previously described [16]. In brief, longitudinally opened arteries or cells cultured on coverslips were fixed in 100% methanol at -20°C for 5 min. After fixation all samples were permeabilized with 0.2% TritonX-100, charges neutralized with 0.5 M NH_4_Cl in PBS for 15 min and blocked in 2% BSA. Subsequently, primary antibodies against Cx40 (Cx40-A; Alpha-Diagnostics lot #175455A8.6; 1/200), Cx37 (Cx37A11-A; Alpha-Diagnostics lot #175859A5-L; 1/50), NFκB (NFκB p65 (A) sc-109; Santa Cruz; 1/100) or Phospho-NFκB (Phospho-NFκB (S536)(93H1); Cell Signaling; 1/100) in blocking solution were applied overnight at 4°C. An Alexa Fluor 488 fluorochrome-conjugated goat anti-rabbit antibody (Life Technologies A11034; 1/5000) was used for signal detection. Cytoplasm and nucleic acids were counterstained with 0.003% Evans Blue (Sigma) and 1/20000 DAPI (Invitrogen), respectively. For eGFP signal detection, arteries were fixed with 4% PFA for 20 min at RT and processed for microscopy. Samples were mounted with Vectashield (Vector Laboratories) and analyzed with a confocal microscope (LSM510-Meta, Zeiss) using LSM AIM software, or an epifluorescent microscope (Axioskop-2, Zeiss) equipped with an AxioCam color CCD camera using Zeiss Axiovision 4.6 software. Quantification of Cx40 immunosignal was performed using ImageJ software.

### Western blot

Cell cultures were rinsed with PBS, pH=7.4, and lysed in RIPA buffer as previously described [15]. After protein concentration quantification with a Micro BCA protein assay kit (Thermo Scientific), an equal quantity of protein was separated by SDS-PAGE and transferred to PVDF-membrane (Immobilon Millipore). After 2 hours blocking with 5% milk and 1% Tween in PBS, the membrane was exposed to primary antibodies (in blocking solution) detecting Cx40 (Cx40-A; Alpha-Diagnostics lot #175455A8.6; 1/500), NFκB (NFκB p65 (A) sc-109; Santa Cruz; 1/1000), P-NFκB (Phospho-NFκB (S536)(93H1); Cell Signaling; 1/1000), IκBα (IκBα L35A5; Cell Signaling; 1/1000), P-IκBα (Phospho-IκB-α (Ser32)(14D4); Cell Signaling; 1/1000) and GAPDH (Millipore MAB374; lot #2388833; 1/30000) as loading control. Revelation was performed by incubating the membrane for 1 hour at RT with secondary horseradish peroxidase-conjugated antibodies (Jackson Immunoresearch; 1/5000) and followed by ECL detection (Millipore) using the Fuji LAS3000 (Fujifilm) and ImageQuant LAS 4000 software. Band intensities were thereafter quantified using ImageJ software.

### Dye coupling

Dye transfer experiments on HeLa cells were performed as previously described [37]. In short, HeLa cells were cultured in 35-mm dishes until confluence. Microelectrodes were pulled from borosilicate glass capillaries (WPI) using a pc-10 electrode puller (Narishige). The electrode was backfilled with 4% Lucifer Yellow (Invitrogen) dissolved in 150 mM LiCl buffered to pH 7.2. Subsequently, the electrode was introduced into one HeLa cell and the dye was allowed to diffuse for 3 min or for 5 min in a separate set of experiments. Fluorescent cells were immediately counted using an inverted TMD-300 microscope (Nikon) equipped with a 40x phase 3 dark medium objective with numerical aperture of 0.7 (Zeiss) and a Lucifer Yellow filter (Zeiss).

### Statistical analysis

Results are presented as mean ± SEM. Unpaired Student's t-tests or one-way ANOVAs were used to compare differences between groups. Differences with a *P* < 0.05 were considered statistically significant; *, P < 0.05; **, P < 0.01; ***, P < 0.001; ****, P < 0.0001.
